# 全腔镜下四孔单向式与三孔法解剖性肺叶切除治疗早期非小细胞肺癌的临床回顾性研究

**DOI:** 10.3779/j.issn.1009-3419.2018.08.02

**Published:** 2018-08-20

**Authors:** 虹 郭, 亚利 刁, 黄新 范, 清泉 罗

**Affiliations:** 1 225000 扬州，扬州大学附属医院扬州市第一人民医院胸外科 Department of Thoracic Surgery, Affiliated to Hospital of Yangzhou University, Yangzhou 225000, China; 2 200240 上海，上海交通大学附属胸科医院胸外科 Department of Thoracic Surgery, Affiliated to Hospital of Shanghai Jiao Tong University, Shanghai 200240, China

**Keywords:** 胸腔镜, 肺叶切除术, 单向式, Thoracoscope, Pulmonary ligament, Monophasic

## Abstract

**背景与目的:**

胸腔镜肺叶切除加纵隔淋巴结清扫术已被认为是早期肺癌手术治疗的标准术式之一。经过二十余年的发展，胸腔镜肺叶切除术的可靠性和微创性已达成共识。目前，胸腔镜肺叶切除术切口选择多样，在三孔基础上逐渐演变成四孔、两孔或单孔作为手术入路。本研究旨在探讨全腔镜下四孔单向式解剖性肺叶切除并纵膈淋巴结清扫术治疗非小细胞肺癌的临床应用价值。

**方法:**

回顾性分析2015年3月-2016年7月扬州大学附属医院扬州市第一人民医院胸外科全腔镜下解剖性肺叶切除并纵隔淋巴结清扫治疗早期外周型非小细胞肺癌临床资料。术前临床诊断为早期外周型非小细胞肺癌。术前按照住院号奇偶数随机分为四孔单向式组（实验组），三孔法组（对照组）。依据纳入及排除标准，最终实验组39例，对照组34例，其中男性36例，女性37例；年龄38岁-84岁。比较两组平均手术时间、平均术中出血量、淋巴结清扫组数、术后平均引流量、平均拔管时间、术后并发症，进行统计学分析。

**结果:**

两组患者均顺利完成手术，术后无死亡。两组平均术中出血、淋巴结清扫组数、术后平均引流量、平均拔管时间、术后并发症差异无统计学意义。四孔单向式组较三孔组平均手术时间缩短，差异有统计学意义（*P* < 0.05）。

**结论:**

全腔镜下四孔单向式手术安全性及疗效满意，术中操作流畅，缩短了手术进程，值得临床推广。

## Abstract (en)

**Background and objective:**

Thoracoscopic lobectomy combined with mediastinal lymph node dissection has been considered as one of the standard surgical procedures for early lung cancer. After 20 years of development, thoracoscopic lobectomy has reached a consensus on reliability and minimally invasive. At present, thoracoscopic lobectomy has a variety of incisions, which gradually evolve into four holes based on three holes, and two or one hole as the operative approach. The aim of this study was to evaluate the clinical value of four-hole unilateral dissecting lobectomy and mediastinal lymph node dissection in the treatment of non-small cell lung cancer (NSCLC). The aim of this study was to investigate the clinical value of anatomical lobectomy with mediastinal lymphadenectomy under four-hole completely video-assisted thoracoscopic surgery (C-VATS) in the treatment of non-small cell lung cancer.

**Methods:**

The patients undergoing lobectomy with mediastinal lymphadenectomy for NSCLC were identified in the Department of Thoracic Surgery, Yangzhou First People's Hospital, Yangzhou University from March 2015 to July 2016. Preoperative clinical diagnosis of peripheral-type early NSCLC. The patients were randomly divided into four-hole monophasic group (experimental group) and three-hole group (control group) according to the number of hospitalization before surgery. According to inclusion and exclusion criteria, the 39 cases assign in experimental group and 34 cases in the control group, including 36 males and 37 females; aged 38 to 84 years. The mean operation time, average blood loss, lymph node dissection group, average drainage, average extubation time and postoperative complications were compared between the two groups for statistical analysis.

**Results:**

The two groups of patients were successfully completed surgery, no death after surgery. Mean bleeding in the two groups, the number of lymph node dissection group, the average postoperative drainage, the average time of extubation, postoperative complications, with no significant difference. The average operation time of the four-hole unidirectional group was shorter than that of the three-hole group. The difference was statistically significant (*P* < 0.05).

**Conclusion:**

The safety and efficacy of a four-hole one-way operation under VATS are satisfactory. The operation is smooth during operation, which shortens the course of operation and deserves the clinical promotion.

非小细胞肺癌（non-small cell lung cancer, NSCLC）已成为全球范围内癌症死亡的首要原因^[1]^。我国是世界第一肺癌大国，肺癌的死亡率上升速度最快、增长幅度最大^[2]^。肺癌预后较差，其5年生存率仍在15%左右^[3]^。Ⅰ期NSCLC首选治疗为肺叶切除加肺门纵隔淋巴结清扫术，其5年生存率可达60%-70%。Ⅱ期NSCLC行手术为主的综合治疗，5年生存率可达35%-40%，由此可见，外科手术仍是早期肺癌首选和最有效的治疗方法^[4]^。全腔镜下肺叶切除术较传统开放手术有着相同的肿瘤切除效果，同时具有手术创伤小、术后疼痛轻、恢复快、切口美观等优点^[5]^，已广泛应用于临床。

## 1 资料与方法

### 1.1 一般资料

收集扬州市第一人民医院自2015年3月-2016年7月完成全腔镜下早期外周型NSCLC根治术资料，依据纳入及排除标准，最终全腔镜下四孔单向式肺叶切除（实验组）39例，其中右上肺叶15例，右中3例，右下9例，左上8例，左下4例，肿瘤大小（2.04±1.06）cm。全腔镜下三孔法肺叶切除（对照组）34例，其中右上肺叶7例，右中3例，右下7例，左上11例，左下6例，肿瘤大小（2.42±0.67）cm。患者的一般情况包括不同肺叶切除比例及肿瘤大小比较无统计学差异（*P* > 0.05）。

### 1.2 入组标准

#### 1.2.1 纳入标准

（1）术前细胞学诊断为早期外周型NSCLC；（2）肿瘤大小≤3 cm；（3）具备肺叶切除条件；（4）性别、基础疾病、年龄不限。

#### 1.2.2 排除标准

（1）术中发现全胸膜腔粘连，无法胸腔镜下完成需中转开胸；（2）术中因麻醉因素、解剖或操作因素需要中转开胸。

### 1.3 治疗方法

#### 1.3.1 术前准备

两组患者术前均雾化吸氧，指导心肺功能锻炼，控制基础疾病，停止吸烟至少1周。术前常规完成胸部CT平扫及增强、气管镜、头颅磁共振成像（magnetic resonance imaging, MRI）、全身骨显像、心脏彩超、心电图、心电图运动实验、全腹部CT或彩超、常规肺功能及弥散功能、血气分析等检查，排除远处转移，评价心肺功能。

#### 1.3.2 四孔单向式各肺叶解剖性切除

四孔法打孔的位置并不固定，主要依据患者的体型，叩诊膈肌位置的高低，肋间隙的宽窄，肺裂及肺门的位置确定打孔的布局。一般而言，上叶切除时观察孔位置在叩诊膈肌最高位，腋前线第7肋间隙交界处。高位操作孔一般做得稍大，约3 cm-4 cm，孔正对肺门或根据水平裂确定，一般选择腋前线第3或第4肋间隙交界处，单独进出吸引器或电凝勾，同时也是肺叶标本进出的孔道。第2操作孔选择在腋后线肩胛下角下方两横指处，约为第6或第7肋间隙，当肋间隙狭窄时可以往腋中线方向移动，术中用于牵拉肺叶。低位操作孔选择在腋中线附近，单独进出吸引器或电凝勾。中叶或下叶切除时观察孔位于腋中线第7肋间隙附近，其它打孔位置相仿。

右上肺叶切除：牵拉肺叶，使肺门结构与躯干长轴近似垂直关系。游离肺门周围纵隔胸膜。由表及里，由前至后，按照上肺静脉→上肺动脉各分支→上叶气管顺序处理各解剖结构。

右中肺叶切除：牵拉肺叶，使肺门结构与躯干长轴近似垂直关系。游离肺门周围纵隔胸膜。按照中叶肺静脉→中叶气管→中叶动脉顺序处理各解剖结构。

右下肺叶切除：牵拉肺叶，使肺门结构与躯干长轴近似垂直关系。游离下肺韧带，解剖肺门前方纵隔胸膜，按照下肺静脉→下肺气管→下肺动脉顺序处理各解剖结构。

左上肺叶切除：牵拉肺叶，使肺门结构与躯干长轴近似垂直关系。游离肺门周围纵隔胸膜。由表及里，由前至后，按照上肺静脉→上叶气管→上肺动脉各分支顺序处理各解剖结构。

左下肺叶切除：牵拉肺叶，使肺门结构与躯干长轴近似垂直关系。游离下肺韧带，解剖肺门前方纵隔胸膜，按照下肺静脉→下肺气管→下肺动脉顺序处理各解剖结构。

#### 1.3.3 三孔法各肺叶解剖性切除

肺裂发育良好时，首先打开肺裂，血管鞘膜，分别处理血管和支气管，具体方法与开放手术相同。肺裂发育不良或不发育时则行单向操作，具体方法同单向式操作。

#### 1.3.4 术中系统性淋巴结清扫

两组患者均在全腔镜下系统性清扫肺叶内、叶间、肺门和纵隔淋巴结。左侧常规清扫第4、5、6、7、8、9、10、11、12组淋巴结，右侧常规清扫第2、4、7、8、9、10、11、12组淋巴结。

### 1.4 统计学软件

采用SSPS 17.0软件对数据进行处理，计数资料采用率（%）表示，组间比较采用χ^2^检验；计量资料采用均数±标准差（Mean±SD）表示，组间比较采用*t*检验，以*P* < 0.05为差异有统计学意义。

## 2 结果

### 2.1 术后早期结果

两组患者均顺利完成手术，术中无中转开胸。术后均恢复顺利，无支气管胸膜瘘，无死亡。实验组术后出现2例乳糜胸，1例反复漏气肺复张不良，对照组术后出现3例乳糜胸，2例反复漏气肺复张不良；并发症发生比例无统计学差异。两组术中平均出血量、平均淋巴结清扫组数、平均拔管时间比较，无统计学差异。两组平均手术时间比较，差异有明显统计学意义（*P* < 0.05），实验组手术更加快捷，术中更加流畅，见表 2。

**1:** 两组患者基本资料比较 The basic data of patients of two groups

Characteristics	Experimental group (*n*=39)	Control group (*n*=34)	*χ*^2^	*t*	*P*
Gender (Male/Female)	19/20	17/17	0.011		0.912
Age (Mean±SD, yr)	60.76±9.12	63.88±8.34		1.512	0.134
Tumor size (Mean±SD, cm)	2.04±1.06	2.42±0.67		1.186	0.066
Different lung			3.707		0.447
Upper-right	15	7			
Middle-right	3	3			
Low-right	9	7			
Upper-left	8	11			
Left-lower	4	6			

**2:** 两组患者观察指标比较（Mean±SD） The observed indicator data of patients of two groups (Mean±SD)

Characteristics	Experimental group (*n*=39)	Control group (*n*=34)	*χ*^2^	*t*	*P*
Postoperative complications	2	3	0.533		0.659
Bleeding volum (mL)	111.02±75.24	141.17±75.34		1.707	0.092
Lymph node dissection	4.58±0.81	4.70±1.21		0.483	0.630
Extubation time (d)	5.48±4.22	7.35±5.52		1.93	0.058
Postoperative drainage (mL)	1, 162.07±1, 171.16	1, 604.61±944.47		1.759	0.082
Tumor size (cm)	2.04±1.06	2.42±0.67		1.186	0.066
Operative time (min)	153.07±54.80	180.14±50.21		2.188	0.031

### 2.2 术后病理及分期

术后病理：非典型腺瘤样增生2例，原位癌6例，鳞状细胞癌8例，腺癌56例，腺鳞癌1例。术后分期：癌前病变2例；Tis 6例；Ⅰa期51例；Ⅰb期10例；Ⅱb期3例；Ⅲa期1例；见表 3。

**3:** 术后病理类型及分期 Pathological types and stages

Index	*n*	Percent (%)
Pathological type		
Atypical adenomatous hyperplasia	2	2.73
Carcinoma *in situ*	6	8.21
Epidermoid carcinoma	8	10.95
Adenocarcinoma	56	76.71
Adenosquamous carcinoma	1	1.36
Total	73	100.00
Pathological stage		
Pre-malignant neoplasm	2	2.73
Tis	6	8.21
Ⅰa	51	69.86
Ⅰb	10	13.69
Ⅱb	3	4.10
Ⅲa	1	1.36
Total	73	100.00

## 3 讨论

1994年McKenna等^[6]^首次报道胸腔镜肺叶切除加纵隔淋巴结采样治疗早期NSCLC这一术式。经过20余年的发展，全胸腔镜手术的安全性及可行性已被广泛认可，国内外胸外科医生已将这一术式广泛应用于临床。文献^[7, 8]^报道了全胸腔镜下治疗早期NSCLC在治疗效果上等甚至优于传统开胸手术的结果。2013年美国国家癌症治疗指南（National Comprehensive Cancer Network, NCCN）则把胸腔镜肺叶切除术作为早期NSCLC的推荐手术方式^[9]^。

全腔镜下肺叶切除的切口选择多样。国内外胸外科医生可以选择4孔、3孔、2孔甚至单孔作为手术入路^[10-12]^。手术方式上，卜梁等^[13]^提出了“王氏”方法，肺裂发育良好可经叶间裂分别处理血管和支气管，如遇到叶间裂分化不全者，先处理肺静脉，再切断支气管，最后分别处理肺动脉各分支，然后将肺叶放回适当位置，沿分裂不全的肺叶裂处用内镜直线缝合切开器将肺叶切除。刘伦旭等^[14]^提出了“单向式”法，正对操作孔下行肺叶切除操作，对需切除肺叶在肺门部位软组织内由最表浅的结构开始解剖，依次暴露、离断，只沿一个方向上逐渐深入，最后处理肺裂，不需来回、上下翻转肺叶；切除上、中叶时采取从前向后单方向推进；切除下叶时为从下向上单方向推进。黄佳等^[15]^提出了“四孔单向式”法，术中使用“罗氏”器械，器械通过独立的孔道进出，单向操作，治疗效果及安全性满意，同时大大提高手术流畅程度及淋巴结清扫程度。支修益^[16]^认为全腔镜下肺叶切除术只是改变了手术入路，而肺癌的外科治疗效果并未因此而改变，应根据各地胸腔镜器械装备的具体情况，手术者技术培训经历和掌握技术的熟练程度，以及地区患者不同的经济承受能力等因素，发展出了各自独具特色的胸腔镜肺叶切除手术方式。我科室全腔镜下肺叶切除术起步较晚，根据自身特点采用传统“三孔”及“四孔单向式”这两种方法。本次研究我们发现两种方式的全腔镜下肺叶切除术，平均术中出血、平均术后引流量、平均拔管时间、术后并发症及平均淋巴结清扫组数并无统计学差异。而平均手术时间有明显统计学差异，四孔单向操作手术时间缩短，过程更加流畅。分析以下因素影响到手术进程。

### 3.1 操作空间及角度的影响

文献^[17]^认为开展此项技术，术者应具有熟练胸腔镜操作技术和丰富开胸肺叶切除手术经验的专业医师。我科室三孔全腔镜下肺叶切除术多由开胸手术经验丰富的医师开展，熟悉的解剖及操作步骤使得此项技术得以顺利开展。三孔法副操作孔一般选择在腋后线与肩胛下线第6、第7或第8肋间。后肋间隙较前肋间隙狭窄，通过副操作孔进行操作存在一定空间及角度限制^[18]^。杨博等^[19]^在其研究中指出8例患者由于解剖因素及操作角度不当而导致肺动脉出血并中转开胸。李运等^[20]^认为为了避免术中肺静脉损伤，应注意正确的处理血管顺序，尽可能使处理血管的角度最佳，保证切开缝合器的砧板能够以最佳角度通过血管后壁。为了可靠地处理血管、气管及肺裂，寻找器械及直线切割器进出的最适角度及空间，术中不可避免地要对肺组织进行多次翻动，对组织进行更多空间上的游离，打通隧道，突破解剖障碍。四孔单项操作在腋中线与肩胛下线间有两个副操作孔，我们称之为高位操作孔及低位操作孔，高位操作孔主要往上牵拉肺组织，使得静脉动脉气管这三大结构长轴与纵膈平面保持垂直状态，解剖仍然遵循由浅入深的原则，无需反复翻动肺叶，只需适当调整就可满足解剖要求。但是术者需要重新认识解剖结构的关系，改变操作习惯。低位操作孔位置更低，多一个操作孔不仅仅是操作空间上的便利与多选择，当解剖靶区不变的情况下，低位操作孔提供了更佳平缓的角度，使得器械尤其是直线切割器的进出更佳稳妥、顺畅，在保证手术安全的同时，提高了手术速度，使得手术更加流畅。

### 3.2 肺裂发育不全的影响

传统三孔法多数习惯于行走肺裂解剖并显露肺动脉各分支^[21]^。而肺裂发育不全或者不发育使得这一操作更加困难与耗时。肺裂发育情况也是胸腔镜解剖性肺切除手术中转开胸的独立危险因素之一^[22]^。而单向沿一个方向推进，由表及里，依次游离，操作中避开了解剖叶间裂，不需在发育全的肺裂中游离肺动脉，最后处理肺裂，用切割缝合器离断非常容易^[23]^，这无疑简化了手术操作，加快了手术进程，手术更加安全。

### 3.3 观察孔及主操作孔的改良

下肺叶及中叶切除时，观察孔的位置仍就选择腋中线，这与三孔操作类似。在上叶操作时，四孔法观察孔则位于腋前线，具体肋间隙则根据患者胸廓形态，叩诊膈肌位置来确定。主操作孔则根据水平裂变化，肺门位置而决定。操作孔正对肺门，同时兼顾上纵膈，一般位于腋前线第3或第4肋间隙。主操作孔及观察孔的改良是为了配合单向操作，使得肺门结构、上纵隔以及肺动脉分支游离时视野更加清晰，稍微改变肺叶方向就可完成解剖操作，而不必大块翻动肺叶。增加的孔道有利于器械的单独进出，这又避免了器械间的相互影响。游离解剖切断可以一气呵成。

总之，四孔单向式手术安全性及疗效满意，术中操作简化而流畅，缩短了手术进程，值得临床推广。
